# Identification of differentially expressed genes by single‐cell transcriptional profiling of umbilical cord and synovial fluid mesenchymal stem cells

**DOI:** 10.1111/jcmm.14891

**Published:** 2019-12-17

**Authors:** Zhaofeng Jia, Shijin Wang, Qisong Liu

**Affiliations:** ^1^ Department of Osteoarthropathy and Institute of Orthopedic Research Shenzhen People's Hospital The Second Clinical Medical College of Jinan University and the First Affiliated Hospital of Southern University of Science and Technology Shenzhen China; ^2^ Department of Orthopaedics Taian City Central Hospital Taian China; ^3^ Institute for Regenerative Medicine Texas A&M Health Science Center College of Medicine Temple TX USA

**Keywords:** DEGs, hSF‐MSCs, hUC‐MSCs, ScRNA‐seq, subpopulations

## Abstract

The purpose of this study was to measure the heterogeneity in human umbilical cord–derived mesenchymal stem cells (hUC‐MSCs) and human synovial fluid–derived mesenchymal stem cells (hSF‐MSCs) by single‐cell RNA‐sequencing (scRNA‐seq). Using Chromium™ technology, scRNA‐seq was performed on hUC‐MSCs and hSF‐MSCs from samples that passed our quality control checks. In order to identify subgroups and activated pathways, several bioinformatics tools were used to analyse the transcriptomic profiles, including clustering, principle components analysis (PCA), t‐Distributed Stochastic Neighbor Embedding (t‐SNE), gene set enrichment analysis, as well as Gene Ontology (GO) and Kyoto Encyclopedia of Genes and Genomes (KEGG) analyses. scRNA‐seq was performed on the two sample sets. In total, there were 104 761 163 reads for the hUC‐MSCs and 6 577 715 for the hSF‐MSCs, with >60% mapping rate. Based on PCA and t‐SNE analyses, we identified 11 subsets within hUC‐MSCs and seven subsets within hSF‐MSCs. Gene set enrichment analysis determined that there were 533, 57, 32, 44, 10, 319, 731, 1037, 90, 25 and 230 differentially expressed genes (DEGs) in the 11 subsets of hUC‐MSCs and 204, 577, 30, 577, 16, 57 and 35 DEGs in the seven subsets of hSF‐MSCs. scRNA‐seq was not only able to identify subpopulations of hUC‐MSCs and hSF‐MSCs within the sample sets, but also provided a digital transcript count of hUC‐MSCs and hSF‐MSCs within a single patient. scRNA‐seq analysis may elucidate some of the biological characteristics of MSCs and allow for a better understanding of the multi‐directional differentiation, immunomodulatory properties and tissue repair capabilities of MSCs.

## 1 INTRODUCTION

Mesenchymal stem cells (MSCs) can differentiate into bone, cartilage and fat cells, which play important roles in development, homeostasis, post‐natal growth, repair and regeneration.^1^, ^2^ Because of their ability to self‐renew with a high proliferation rate, MSCs are a common source of stem cells in clinical applications to regenerate damaged organs and tissues.^3^, ^4^ Numerous studies indicate that the major sources for MSCs in the clinical setting are adipose tissue and bone marrow; however, these resources are limited because there are strict donor requirements.^5^, ^6^, ^7^ Therefore, alternative sources obtained from neonatal or primitive tissues, such as the amnion, placenta, synovial fluid and umbilical cord, have been explored.^2^, ^8^, ^9^, ^10^

The umbilical cord (UC) is an attractive source of MSCs as it can be obtained by non‐invasive methods without harm to mothers or their children.^11^ The UC possesses immunosuppressive activity and produces an abundance of MSCs.^12^, ^13^ UC‐derived MSCs (UC‐MSC) are one type of multipotent adult stem cell, which has the potential to differentiate into various cell types, thereby making these cells a possible resource for cell‐based therapies. Human umbilical cord‐derived mesenchymal stem cells have some characteristics in common with MSCs obtained from adipose tissue and bone marrow, including a fibroblastoid morphology and a similar set of surface proteins, as well the ability to differentiate into different cell types.^14^, ^15^

Previous studies have shown that MSCs also exist in synovial fluid (SF).^16^, ^17^ In the presence of an injury or osteoarthritis, the number of MSCs from SF increases significantly in order to help recruit mesenchymal progenitor cells to promote spontaneous healing and restore homeostasis.^17^ SF‐derived MSCs (SF‐MSCs) are a viable option for syngeneic transplantation for cartilage regeneration.^18^, ^19^ SF‐MSCs are ideal for clinical applications because SF can be obtained arthroscopically without the donor undergoing invasive surgery.

In recent years, single‐cell genomics has become an incredibly powerful tool to help uncover the genetic structure and population dynamics of unicellular organisms,^20^, ^21^, ^22^, ^23^ as well as cancer cells,^24^ and has provided insight into the developmental lineages^25^ in multicellular organisms. Single‐cell RNA‐sequencing (scRNA‐seq) can be used to analyse differences in the transcriptome of various cells,^26^, ^27^ discover novel cell types and provide insights into the regulatory networks that function in ontogenetic development.^28^ scRNA‐seq is an efficient method for analysing changes in gene expression, and it has been performed successfully in many different tissue types.^29^, ^30^, ^31^ In order to uncover information about the subpopulations that exist in MSCs and analyse the differentially expressed genes (DEGs) of these subgroups, we used scRNA‐seq to perform transcriptomic profiling in hUC‐MSCs and hSF‐MSCs. Furthermore, using clustering, principle components analysis (PCA), t‐Distributed Stochastic Neighbor Embedding (t‐SNE), gene set enrichment analysis, Gene Ontology (GO) and Kyoto Encyclopedia of Genes and Genomes (KEGG) analyses, we were able to identify subgroups and activated pathways within these populations of MSCs.

## 2 MATERIALS AND METHODS

### 2.1 Ethics statement

This study was conducted using protocols approved by the Ethical Committee of the Shenzhen People's Hospital. Informed consents were obtained from all participants.

### 2.2 Isolation and culture of hUC‐MSCs

Under sterile conditions, UC units (8‐10 cm) were collected from the puerpera of full‐term deliveries and were immediately saved in cold saline (0°C). The blood vessels and outer membrane were removed by surgical blades, and the Wharton jelly (WJ) tissue was cut using eye scissors. The minced tissues were then placed in a 10‐cm culture dish at 1‐cm intervals and were maintained in culture medium (MesenGro Medium supplemented with 10% FBS, 1% penicillin‐streptomycin and 10% MesenGro Supplement) at 37°C with 5% CO_2_ and 90% RH. After 48 hours, the medium was removed to eliminate non‐adherent cells and replaced with fresh medium. The complete culture medium was changed every 3 days. We selected distinct cell subpopulations, and we assumed that these subpopulations were efficient and sustainable. Colonies smaller than 2 mm in diameter were ignored. The clustered hUC‐MSCs were digested with trypsin and resuspended with complete culture medium at a density of 2.0 × 10^4^ cells/cm^2^ into a 25‐mm^2^ vented culture bottle. The CD90^+^ hUC‐MSCs were collected by immunomagnetic beads and were identified under more stringent measures. After approximately three generations, the hUC‐MSCs were sterilely obtained to prepare a monoplast suspension of more than 6.0 × 10^5^ cells, with a survival rate >90% and cell diameter <30 μm. The hUC‐MSCs samples were then sent to GENERGY BIO (Shanghai, China) for scRNA‐seq analysis.

### 2.3 Isolation and culture of hSF‐MSCs

The samples were collected during arthroscopic procedures from patients suffering from an intra‐articular ligament injury of the knee joint. Isotonic saline solution was injected into the joint, the knee was moved several times, and then, SF (50‐100 mL) mixed with saline solution was collected in γ‐sterilized centrifuge tubes. Within 1‐4 hours, the fluid was filtered with a cell strainer (40 μm nylon) to remove debris. The filtered fluid was gathered in γ‐sterilized centrifuge tubes and centrifuged at 405 *g* for 10 minutes at room temperature. The cell pellet was resuspended in culture medium (MesenGro Medium supplemented with 10% FBS, 1% penicillin‐streptomycin and 10% MesenGro Supplement) and plated in 100‐mm dishes after centrifugation. After 48 hours, the medium was withdrawn to remove non‐adherent cells and replaced with fresh medium. The complete culture medium was changed every 3 days. We selected distinct cell subpopulations and assumed that these units were efficient and sustainable. The colonies smaller than 2 mm in diameter were discarded using a cell scraper (Corning Inc). Then, the distinct cell subpopulation was digested in cloning cylinders (Sigma‐Aldrich) and used to inoculate a new dish as passage 1. Passage 3 (P3) cells were used for the scRNA‐seq analysis.

### 2.4 The scRNA‐seq analysis

**Figure 1:**
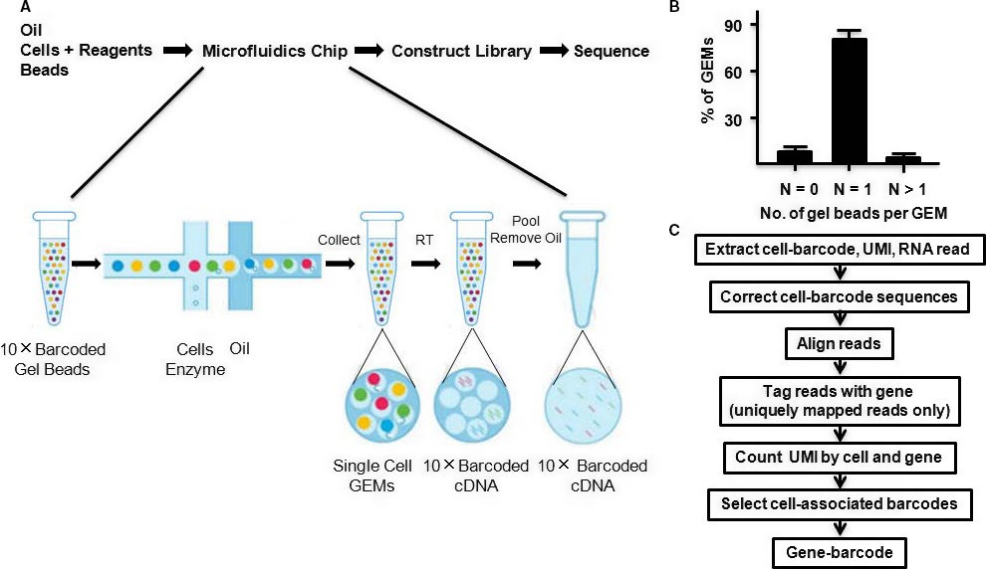
10 × Genomics single‐cell technology enables the profiling of RNAs from thousands of single cells simultaneously. Cells were combined with reagents in one channel of a chip. Reverse transcription took place inside each GEM, after which cDNAs were pooled to perform amplification and library construction in bulk. Gel beads loaded with primers and barcoded oligonucleotides were first mixed with cells and reagents, and subsequently mixed with oil‐surfactant solution at a microfluidic junction. Single‐cell GEMs were collected in the GEM outlet. Finished library molecules consisted of Illumina adapters and sample indices, which allowed for pooling and sequencing of multiple libraries on a next‐generation short read sequencer

The hUC‐MSCs and hSF‐MSCs samples were sent to GENERGY BIO for scRNA‐seq analysis (Figure 1) by following previously published protocols.^29^, ^31^ Briefly, cell counting was performed using a Countess^®^ II Automated Cell Counter and cell concentration was adjusted to 1.0 × 10^6^ cells/mL. Cellular suspensions were placed on a Chromium™ Single‐Cell Instrument (10 × Genomics) to obtain single‐cell Gel bead in EMulsion (GEM). ScRNA‐seq libraries were prepared using the Chromium™ Single‐Cell Bead and Library Kit. cDNA was sheared to 200 bp using a Covaris M220 (Covaris). Sequencing libraries were constructed using the Chromium™ Single‐Cell Library Kit, following these steps: end repair and A‐tailing, adapter ligation, post‐ligation cleanup with SPRIselect, and sample index PCR and cleanup. The barcode sequencing libraries were quantified using qPCR.

The Cell Ranger Single‐Cell Software Suite was used to perform sample demultiplexing, barcode processing and single‐cell gene counting. First, sample demultiplexing was performed to generate FASTQs for the paired‐end Read1 and Read2. Second, Chromium™ barcodes and unique molecular identifiers (UMIs) were filtered. Third, PCR was marked if read pairs matched with a barcode sequence, a UMI tag and a gene ID. Cell barcodes were determined based on the distribution of UMI counts. The number of reads that provided valid information was determined based on whether the reads had the following four characteristics: valid barcodes, a valid UMI, a cell barcode and the ability to be confidently mapped to exons. Sequencing data could be combined by counting non‐duplicated reads and subsampled to obtain a given number of UMI counts per cell.

The gene‐cell‐barcode matrix was concatenated. Only genes with at least one UMI count detected in at least one cell were used. Unique molecular identifier normalization was performed by first dividing UMI counts, followed by multiplication by the median total UMI counts across all cells. Each gene was normalized such that its mean signal was 0, and standard deviation was 1. Principle components analysis was run on the normalized gene‐barcode matrix. The normalized UMI counts of each gene were used to show expression of a marker in a t‐SNE plot.

To identify genes that were enriched in a specific cluster, the mean expression of each gene was calculated across all cells in the cluster. Each gene from the cluster was then compared to the median expression of the same gene in all other cell clusters. Genes were ranked based on their expression difference, and the top 10 enriched genes from each cluster were selected. For hierarchical clustering, pair‐wise correlation between each cluster was calculated, and centred expression of each gene was used to generate a heat map. Gene Ontology and KEGG term information was downloaded from the UniProtKB database. Both GO and KEGG terms with a *P*‐value < .05 were considered to be significantly enriched.

### 2.5 Statistical analysis

Data are available at http://support.10%D7genomics.com/single-cell/datasets, and the analysis code is available at https://github.com/10%D7Genomics/single-cell-3prime-paper.

## 3 RESULTS

### 3.1 The scRNA‐seq profiles of hUC‐MSCs and hSF‐MSCs by 10 × Genomics

For our scRNA analysis, we obtained 1597 cells and 1259 cells from hUC‐MSCs and hSF‐MSCs samples, respectively (Table 1). The sequencing saturation for hUC‐MSCs and hSF‐MSCs samples was 43.4% and 18.1%, respectively (Table 1). In hUC‐MSCs, 17 317 genes were detected, with a median of 18 304 UMI Counts per cell, and an average of 65 598 reads per cell (Table 1). There were 104 761 163 reads from hUC‐MSCs, of which, 86.2% had valid barcodes (Table 1). After mapping to the human genome, 78.2% of the reads mapped confidently to the transcriptome, with 81.8% of those reads mapping to exonic regions, 5.9% to intronic regions and 2.1% to intergenic regions (Table 1). In hSF‐MSCs, 16 996 genes were detected, with a median of 12 609 UMI Counts per cell, and an average of 52 245 reads per cell (Table 1). There were 6 577 715 reads from hSF‐MSCs, with 70.4% of those reads containing valid barcodes (Table 1). After mapping to the human genome, 60.4% of those reads mapped confidently to transcriptome, with 63.1% of those reads mapping to exonic regions, 8.1% to intronic regions and 2.6% to intergenic regions (Table 1).

**Table 1:** Summary of single cells sequencing by 10 × Genomics

	hUC‐MSCs	hSF‐MSCs
Cells		
Estimated number of cells	1597	1259
Fraction reads in cells	81.2%	77.8%
Mean reads per cell	65 598	52 245
Median genes per cell	3333	2954
Total genes detected	17 317	16 996
Median UMI counts per cell	18 304	12 609
Sequencing		
Number of reads	104 761 163	65 777 15
Valid barcodes	86.2%	70.4%
Reads mapped confidently to transcriptome	78.2%	60.4%
Reads mapped confidently to exonic regions	81.8%	63.1%
Reads mapped confidently to intronic regions	5.9%	8.1%
Reads mapped confidently to intergenic regions	2.1%	2.6%
Sequencing saturation	43.4%	18.1%
Q30 bases in barcode	47.2%	29.6%
Q30 bases in RNA read	89.2%	62.2%
Q30 bases in sample index	89.0%	70.9%
Q30 bases in UMI	92.6%	67.5%
Sample		
Transcriptome		GRCh38
Chemistry		Single Cell 3' v1
Cell ranger version		1.2.0

### 3.2 Subpopulation discovery in hUC‐MSCs and hSF‐MSCs samples

**Figure 2:**
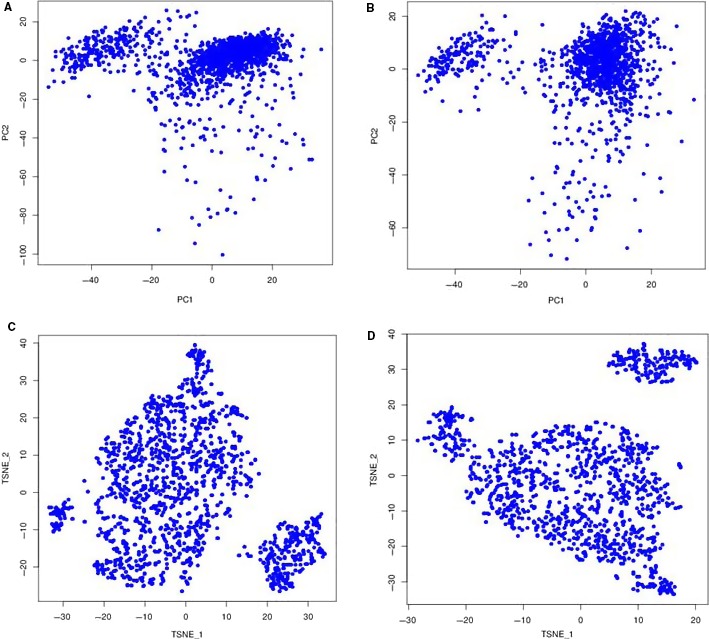
The principle components analysis (PCA) and t‐Distributed Stochastic Neighbor Embedding (t‐SNE) for hUC‐MSCs and hSF‐MSCs. A, PCA for hUC‐MSCs, B, PCA for hSF‐MSCs, C, t‐SNE for hUC‐MSCs, D, t‐SNE for hSF‐MSCs

**Figure 3:**
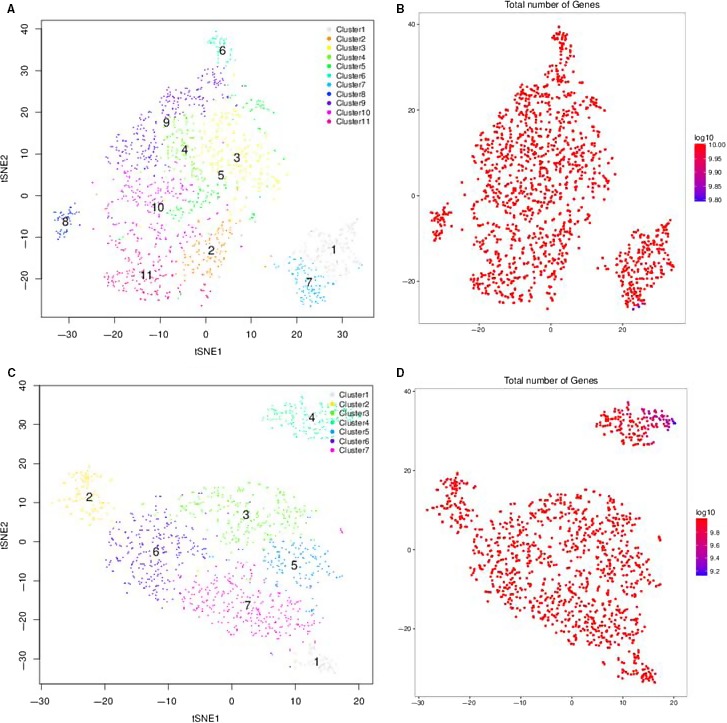
Subpopulation discovery in hUC‐MSCs (A‐B) and hSF‐MSCs (C‐D) by t‐SNE analysis. Based on our PCA and t‐SNE results, there were 11 clusters present in hUC‐MSCs and 7 in hSF‐MSCs

The Chromium™ single‐cell technology can also be used for scRNA‐seq of primary cells. We isolated more than 1000 cells from hUC‐MSCs and hSF‐MSCs. Gene‐cell matrices from hUC‐MSCs and hSF‐MSCs were concatenated, and PCA was performed to reduce dimensionality before performing clustering and t‐SNE analysis. Based on our PCA and t‐SNE results, there were 11 clusters present in hUC‐MSCs and 7 in hSF‐MSCs (Figures 2 and 3).

### 3.3 The DEG profile of hUC‐MSCs and hSF‐MSCs samples

By comparing gene expression profiles between subpopulations in hUC‐MSCs, we identified 533 DEGs in cluster 1 (76 up‐regulated and 457 down‐regulated), 57 in cluster 2 (12 up‐regulated and 45 down‐regulated), 32 in cluster 3 (28 up‐regulated and four down‐regulated), 44 in cluster 4 (41 up‐regulated and three down‐regulated), 10 in cluster 5 (seven up‐regulated and three down‐regulated), 319 in cluster 6 (286 up‐regulated and 31 down‐regulated), 731 in cluster 7 (53 up‐regulated and 678 down‐regulated), 1037 in cluster 8 (424 up‐regulated and 613 down‐regulated), 90 in cluster 9 (89 up‐regulated and one down‐regulated), 25 in cluster 10 (24 up‐regulated and one down‐regulated) and 230 in cluster 11 (31 up‐regulated and 199 down‐regulated) (Tables 2 and 3). In hSF‐MSCs, there were 204 DEGs in cluster 1 (185 up‐regulated and 19 down‐regulated), 577 in cluster 2 (129 up‐regulated and 448 down‐regulated), 30 in cluster 3 (20 up‐regulated and 10 down‐regulated), 577 in cluster 4 (43 up‐regulated and 534 down‐regulated), 16 in cluster 5 (14 up‐regulated and two down‐regulated), 57 in cluster 6 (51 up‐regulated and down down‐regulated) and 35 in cluster 7 (32 up‐regulated and three down‐regulated) (Tables 2 and 4).

**Table 2:** Differentially expressed genes in each cluster of hUC‐MSCs and hSF‐MSCs

hUC‐MSCs	Differentially expressed genes			hSF‐MSCs	Differentially expressed genes		
	Up‐regulated	Down‐regulated	Total		Up‐regulated	Down‐regulated	Total
Cluster 1	76	457	533	Cluster 1	185	19	204
Cluster 2	12	45	57	Cluster 2	129	448	577
Cluster 3	28	4	32	Cluster 3	20	10	30
Cluster 4	41	3	44	Cluster 4	43	534	577
Cluster 5	7	3	10	Cluster 5	14	2	16
Cluster 6	286	31	319	Cluster 6	51	6	57
Cluster 7	53	678	731	Cluster 7	32	3	35
Cluster 8	424	613	1037				
Cluster 9	89	1	90				
Cluster 10	24	1	25				
Cluster 11	31	199	230				

**Table 3:** Differentially expressed genes in each cluster of hUC‐MSCs

hUC‐MSCs	Up‐regulated	Down‐regulated
Cluster 1	COTL1; PRDX1; CAPN2; ATP5B; VIM; ACTB; ACTG1; VDAC1; PGK1; ANXA1	HERPUD1; CYR61; MALAT1; NEAT1; ATP2B1; H3F3B; THBS1; COL1A2; COL1A1; FN1
Cluster 2	TUBA1B; CCT8; ATP5A1; LDHB; VIM; TUFM; C4orf3; TAGLN	FDPS; ATP5B; HSPA5; PDIA3; COL6A1; SAT1; BRI3; CDKN1A; NEAT1; GADD45A; ANKRD1; LINC00152
Cluster 3	CDKN1A; FHL2; ADM; MALAT1; H3F3B; RPL22L1; TNFRSF12A; TGFBI; NUPR1; MEST	MGLL; PPP1R14B; SNA12; IER3
Cluster 4	S100A16; POSTN; TGFBI; SAA1; PELO; BDNF; CTGF; ANKRD1; ACAT2; CTHRC1	PPME1; CITED2; PDLIM1
Cluster 5	DDIT4; INSIG1; HERPUD1; ATP2B1; MALAT1; TGFBI; TAGLN	PTMA; H3F3B; MGST3
Cluster 6	H2AFZ; HMGB1; PTTG1; STMN1; KIAA0101; H;IST1H4C; TUBB4B; HMGB2; SMC4; CCNB1	CDKN2A; IFITM3; C4orf3; TAGLN; GLRX; SAT1; S100A13; SELM; FTL; FTH1
Cluster 7	EEF1A1; COTL1; RPL31; RPL21; VIM; PDIA3; TGFBI; POSTN; TFPI2; CD59	CYR61; MALAT1; NEAT1; NNMT; HERPUD1; TUBA1A; TUBA1B; KRT10; H3F3B
Cluster 8	PDIA3; MALAT1; NEAT1; FN1; COL1A1; COL3A1; MT‐ATP6; MT‐CO2; MT‐CO3; MT‐CO1	TPT1; RPS20; RPL12; RPL29; RPL37; RPS12; RPS15A; RPS8; RPL32; RPL18A
Cluster 9	RPL21; HERPUD1; CYR61; UBC; HSPA5; PCOLCE; MSMO1; ACAT2; TUBA1B; TUBA1A	MALAT1
Cluster 10	S100A16; HMGA1; ANKRD1; COL4A2; COL1A1; NEAT1; MALAT1; NUPR1; DCN; IGFBP7	COL8A1
Cluster 11	GHITM; ARF4; VDAC1; ANXA1; YWHAQ; HSP90AA1; ITM2B; RTN4; CNN3	MEG3; MALAT1; NEAT1; COL6A2; COL6A1; MT‐ATP6; MT‐CO3; FLNA; COL1A1; FN1; STRAP

**Table 4:** Differentially expressed genes in each cluster of hSF‐MSCs

hSF‐MSCs	Up‐regulated	Down‐regulated
Cluster 1	HMGB1; STMN1; H2AFZ; KIAA0101; PTTG1; CKS2; HIST1H4C; TYMS; HMGB2; UBE2C	CDKN2A; FTH1; NUPR1; TGFBI; PAPPA; PSAP; NEAT1; FN1; GAS6; POSTN
Cluster 2	MALAT1; NEAT1; MT‐ND3; MT‐ND2; MT‐ATP6; MT‐CYB; MT‐CO2; MT‐ND4; MT‐CO3; MT‐CO1	TPT1; FTL; FTH1; TMSB4X; RPL29; RPL23A; RPS12; RPL32; RPL12; RPL18A
Cluster 3	FTH1; MGLL; SRPX; CD59; ANXA2; S100A4; PGLDA2; C12orf75	CXCL1; SOX4; NEAT1; MALAT1; FN1; COL1A1; DCN; GJA1; PTGDS; SERPINE1; TUBA1B; CAV1
Cluster 4	FKBP1A; POLR2L; TXN; S100A11; S100A6; YBX1; RPL31; RPLP0; CD59; ACTB	MALAT1; NEAT1; FN1; COL1A1; COL1A2; EMP1; H3F3B; KRT10; CYR61; TUBA1A
Cluster 5	POSTN; GAS6; NDUFA4L2; SCG5; RPL22L1; CXCL3; CXCL1; CXCL8; INSIG1; H3F3B	LMO4; TUBA1B
Cluster 6	DCBLD2; FRMD6; HIF1A; NEAT1; TGFBI; GREM1; ADAMTS1; CCDC80; SERPINE1; PLOD2	LGALS3; B2M; PCOLCE; PPIB; TIMP1; CD63
Cluster 7	NDUFA4L2; TGFBI; PAPPA; S100A4; CAPG; TNFRSF12A; MGLL; LDHA; LGALS3; S100A16	H3F3B; CALM2; TUBA1B

### 3.4 GO function analysis of hUC‐MSCs and hSF‐MSCs samples

**Figure 4:**
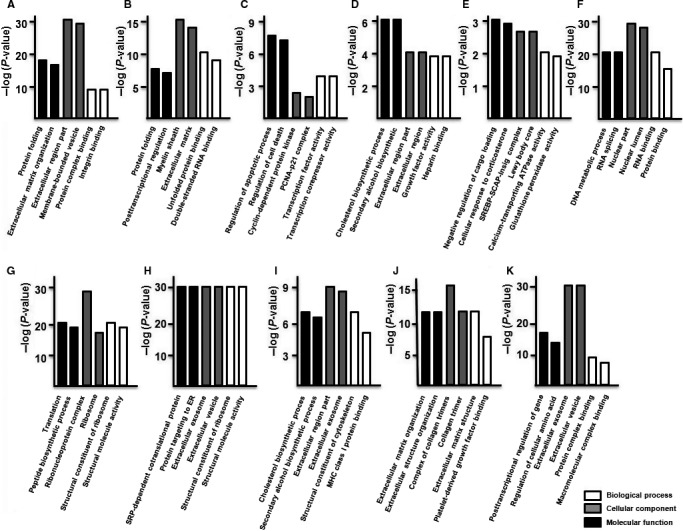
GO function analysis of the DEGs of hUC‐MSCs. GO classification of differentially regulated genes in cluster 1 (A), cluster 2 (B), cluster 3 (C), cluster 4 (D), cluster 5 (E), cluster 6 (F), cluster 7 (G), cluster 8 (H), cluster 9 (I), cluster 10 (J) and cluster 11 (K). The results were separated into three main categories: biological process, cellular component and molecular function

The three main categories for GO function analysis are biological process, cellular component and molecular function. As shown in Figure 4, the DEGs found in hUC‐MSCs were significantly enriched in the following biological processes and molecular functions: cholesterol biosynthesis, calcium‐transporting ATPase activity, corticosterone response, cholesterol biosynthesis, collagen trimer, cyclin‐dependent protein kinase, DNA metabolism, double‐stranded RNA binding, glutathione peroxidase activity, growth factor activity, heparin binding, integrin binding, macromolecular complex binding, MHC class I protein binding, negative regulation of cargo loading, PCNA‐p21 complex, platelet‐derived growth factor binding, protein binding, protein folding, peptide biosynthetic process, protein complex binding, protein targeting to ER, post‐transcriptional regulation of gene expression, regulation of apoptotic process, regulation of cell death, regulation of cellular amino acid metabolism, RNA binding, RNA splicing, ribonucleoprotein complex, secondary alcohol biosynthetic process, SREBP‐SCAP‐Insig complex, SRP‐dependent cotranslational protein, translation, structural molecule activity, transcription factor activity, transcription corepressor activity and unfolded protein binding. Additionally, GO term analysis showed that many of the DEGs function in the extracellular exosome, extracellular matrix, extracellular vesicles, membrane‐bounded vesicles, Lewy bodies, myelin sheath and nucleus, and as structural components in the ribosome and cytoskeleton.

**Figure 5:**
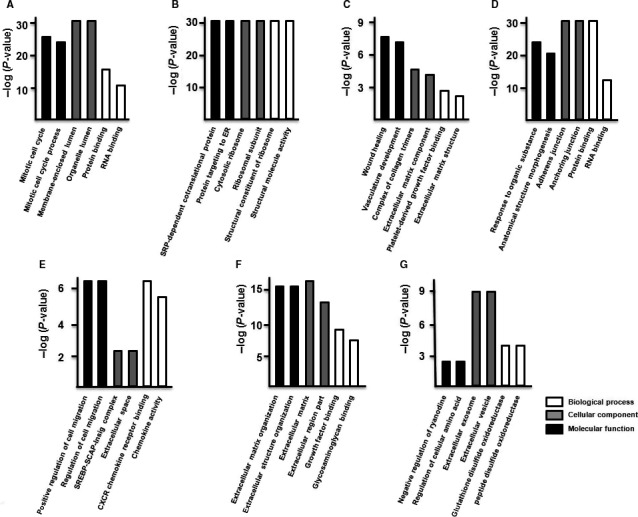
GO function analysis of the DEGs of hSF‐MSCs. GO classification of differentially regulated genes in cluster 1 (A), cluster 2 (B), cluster 3 (C), cluster 4 (D), cluster 5 (E), cluster 6 (F) and cluster 7 (G). The results were separated into three main categories: biological process, cellular component and molecular function

In hSF‐MSCs, the cluster 1 DEGs were primarily associated with mitotic cell cycle control and processes, protein binding and RNA binding, and were enriched in membrane‐enclosed lumen and organelle lumen (Figure 5A). The DEGs of cluster 2 were primarily related to SRP‐dependent cotranslational protein targeting, protein targeting to ER and structural molecule activity, and a significant amount is found in cytosolic ribosomes and ribosomal structures (Figure 5B). The DEGs of cluster 3 function in wound healing, vasculature development, platelet‐derived growth factor binding, formation of collagen trimers and extracellular matrix structure (Figure 5C). The DEGs of cluster 4 were related to responding to organic substances, anatomical structure morphogenesis and protein binding and RNA binding, and many of them are found in adherens junctions and anchoring junctions (Figure 5D). The DEGs of cluster 5 were associated with the regulation of cell migration, SREBP‐SCAP‐Insig complex, CXCR chemokine receptor binding and chemokine activity, with many of the DEGs localizing to the extracellular space (Figure 5E). The DEGs of cluster 6 were primarily related to extracellular matrix organization, extracellular structure organization, growth factor binding and glycosaminoglycan binding (Figure 5F). The DEGs of cluster 7 were primarily associated with the negative regulation of ryanodine, regulation of cellular amino acid, glutathione disulphide oxidoreductase and peptide disulphide oxidoreductase and a significant number of the DEGs localized to extracellular exosome, extracellular vesicle (Figure 5G).

### 3.5 KEGG analysis of hUC‐MSCs and hSF‐MSCs samples

**Figure 6:**
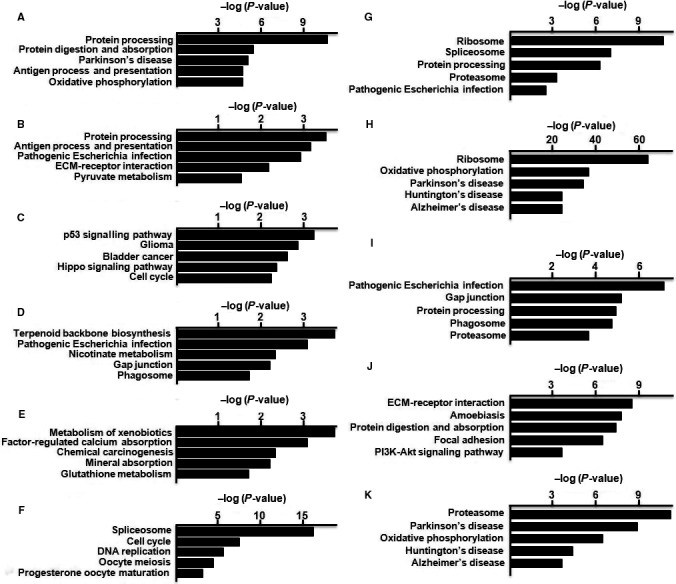
KEGG analysis of the DEGs of hUC‐MSCs. KEGG classification of differentially regulated genes in cluster 1 (A), cluster 2 (B), cluster 3 (C), cluster 4 (D), cluster 5 (E), cluster 6 (F), cluster 7 (G), cluster 8 (H), cluster 9 (I), cluster 10 (J) and cluster 11 (K)

According to KEGG analysis, the DEGs in hUC‐MSC were mainly enriched in Alzheimer's disease, amoebiasis, antigen process and presentation, bladder cancer, cell cycle, chemical carcinogenesis, DNA replication, ECM‐receptor interactions, factor‐regulated calcium absorption, focal adhesions, gap junctions, glioma, glutathione metabolism, Hippo signalling pathway, Hunting's disease, Huntington's disease, xenobiotics metabolism, mineral absorption, nicotinate metabolism, oocyte meiosis, oxidative phosphorylation, p53 signalling pathway, Parkinson's disease, pathogenic Escherichia infection, PI3K‐Akt signalling pathway, progesterone oocyte maturation, protein digestion and absorption, protein processing, pyruvate metabolism, and terpenoid backbone biosynthesis, and function in the phagosome, proteasome, ribosome and spliceosome (Figure 6).

**Figure 7:**
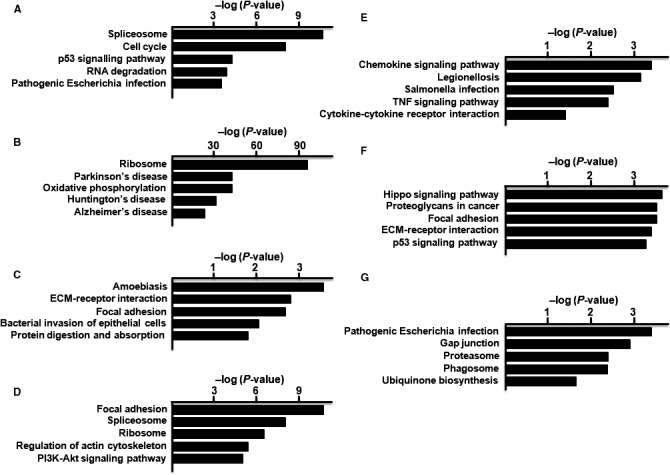
KEGG analysis of the DEGs of hSF‐MSCs. KEGG classification of differentially regulated genes in cluster 1 (A), cluster 2 (B), cluster 3 (C), cluster 4 (D), cluster 5 (E), cluster 6 (F) and cluster 7 (G)

In hSF‐MSCs, the DEGs in cluster 1 were primarily involved in the spliceosome, cell cycle, p53 signalling pathway, RNA degradation and pathogenic *Escherichia* infection (Figure 7A). The DEGs of cluster 2 were associated with the ribosome, Parkinson's disease, oxidative phosphorylation, Huntington's disease and Alzheimer's disease (Figure 7B). The cluster 3 DEGs were primarily related to amoebiasis, ECM‐receptor interactions, focal adhesions, bacterial invasion of epithelial cells and protein digestion and absorption (Figure 7C). Differentially expressed genes from cluster 4 were primarily associated with the spliceosome, ribosome and focal adhesions, as well as regulation of actin cytoskeleton, and PI3K‐Akt signalling (Figure 7D). In cluster 5, the DEGs were primarily connected to chemokine signalling, Legionellosis, *Salmonella* infection, TNF signalling and cytokine‐cytokine receptor interactions (Figure 7E). The DEGs of cluster 6 were primarily associated with Hippo signalling, proteoglycans expressed in cancer cells, focal adhesions, ECM‐receptor interactions and p53 signalling (Figure 7F). The DEGs of cluster 7 were primarily related to the proteasome and phagosome, as well as pathogenic *Escherichia* infection, gap junction formation and ubiquinone biosynthesis (Figure 7G).

## 4 DISCUSSION

In general, MSCs are increasingly being used as a resource for cell‐based therapies in cartilage repair and regenerative medicine.^32^, ^33^ The most effective cell dosages for clinical applications are still unclear; however, it is likely that a large number of MSCs would be needed for both cartilage repair and regenerative medicine. Over the last few years, adipose tissue, bone marrow, synovial fluid and umbilical cord blood have become accessible sources of cells for tissue engineering therapies. Adipose tissue contains MSCs at the highest concentration, and umbilical cord blood can be easily expanded to obtain higher numbers of MSCs.^34^ Many different laboratories have studied the cell morphology, surface markers and differentiation capacity of stem cells from these sources in order to gain a better understanding of the basic biology of various MSCs.^4^, ^15^ In this study, we were able to shed some light on the subgroups present in MSCs and the DEGs within those groups using scRNA‐seq.

Since UC‐MSCs are easily accessible and present fewer ethical problems, they are advantageous as potential resource for cell therapies and clinical applications. Tumorigenesis in UC‐MSCs and UC‐MSC‐derived transplant cells is rarely reported,^34^, ^35^ and UC‐MSCs have the potential to differentiate into a variety of different cell types.^36^, ^37^, ^38^, ^39^ SF‐MSCs provide another source for cell‐based therapies,^16^ although the success rates for SF‐MSCs are varied and the frequency of SF‐MSCs colony development is low.^40^ In this study, all cells isolated from the umbilical cord and synovial fluid exhibited typical MSC characteristics with a fibroblastoid morphology, a multipotential differentiation capability and a typical set of surface proteins.

With scRNA‐seq, we obtained 1597 cells and 1259 cells from hUC‐MSCs and hSF‐MSCs samples, respectively. The sequencing saturation for hUC‐MSCs and hSF‐MSCs samples was 43.4% and 18.1%, respectively. After mapping to the human genome, a majority of the reads could be confidently mapped to exonic regions (81.8% for hUC‐MSC and 63.1% for hSF‐MSC). As shown in Figures 2 and 3, PCA and t‐SNE analysis unveiled 11 clusters in hUC‐MSCs and 7 in hSF‐MSCs. In hUC‐MSC and hSF‐MSC, the total number of genes detected was 17 317 and 16 996, respectively. By comparing gene expression profiles between subpopulations, we identified 533 DEGs in cluster 1 of hUC‐MCSs, 57 in cluster 2, 32 in cluster 3, 44 in cluster 4, 10 in cluster 5, 319 in cluster 6, 731 in cluster 7, 1037 in cluster 8, 90 in cluster 9, 25 in cluster 10 and 230 in cluster 11 (Table 2). In hSF‐MSCs, there were 204 DEGs in cluster 1, 577 in cluster 2, 30 in cluster 3, 577 in cluster 4, 16 in cluster 5, 57 in cluster 6 and 35 in cluster 7 (Table 2).

The DEGs of hUC‐MSCs and hSF‐MSCs were mainly enriched in several biological processes and molecular functions, including cellular response to corticosterone, cholesterol biosynthetic process, cyclin‐dependent protein kinase, DNA metabolic process, extracellular matrix organization, growth factor activity, macromolecular complex binding, negative regulation of cargo loading, protein binding, protein folding, protein complex binding, protein targeting to ER, post‐transcriptional regulation of gene expression, regulation of apoptotic process, regulation of cell death, regulation of amino acid metabolism, RNA binding, RNA splicing, secondary alcohol biosynthetic process, translation, structural molecule activity, transcription factor activity, transcription corepressor activity and unfolded protein binding. The DEGs were localized to particular cellular components including the extracellular exosome, extracellular matrix, extracellular region, extracellular vesicle, membrane‐bounded vesicle, nucleus, nuclear lumen, ribosome and cytoskeleton.

The DEGs of hUC‐MSCs and hSF‐MSCs were mainly enriched in several signalling pathways, including Alzheimer's disease, amoebiasis, antigen processing and presentation, cell cycle, chemical carcinogenesis, DNA replication, ECM‐receptor interactions, factor‐regulated calcium absorption, focal adhesion signalling, gap junction signalling, glioma, glutathione metabolism, Hippo signalling pathway, Huntington's disease, mineral absorption, nicotinate metabolism, oocyte meiosis, oxidative phosphorylation, p53 signalling pathway, Parkinson's disease, pathogenic Escherichia infection, PI3K‐Akt signalling pathway, progesterone oocyte maturation, protein digestion and absorption, protein processing, proteasome signalling, pyruvate metabolism, ribosome, phagosome, spliceosome and terpenoid backbone biosynthesis.

In summary, our work provides information about the subpopulations and DEGs in hUC‐MSCs and hSF‐MSCs, as identified via scRNA‐seq. In addition, our results show that these DEGs are involved in many pathways, such as p53 signalling, gap junction signalling and PI3K‐Akt signalling, that function in regenerating damaged organs and tissues. Though our results are promising and provide important information about MSCs, the characteristics of hUC‐MSCs and hSF‐MSCs, and the functions of the differentially expressed genes in these subpopulations, need to be studied further.

## CONFLICT OF INTEREST

The authors confirm that there are no conflicts of interest.

## AUTHOR CONTRIBUTIONS

The following people designed, performed research and analysed data: Zhaofeng Jia, Shijin Wang, Qisong Liu; Zhaofeng Jia wrote the paper.

## Data Availability

The raw data used for the analyses in this paper have been deposited in the Genome Sequence Archive in BIG Data Center, Beijing Institute of Genomics (BIG), Chinese Academy of Sciences, under accession numbers CRA002294, CRA002294 that are publicly accessible at https://bigd.big.ac.cn/gsa.
